# Concentrations of Mineral in Amniotic Fluid and Their Relations to Selected Maternal and Fetal Parameters

**DOI:** 10.1007/s12011-015-0557-3

**Published:** 2015-11-07

**Authors:** J. Suliburska, R. Kocyłowski, I. Komorowicz, M. Grzesiak, P. Bogdański, D. Barałkiewicz

**Affiliations:** Department of Human Nutrition and Hygiene, Poznan University of Life Sciences, ul. Wojska Polskiego 31, 60-624 Poznan, Poland; PreMediCare New Med Medical Centre, ul. Drużbickiego 13, 61-693 Poznan, Poland; Department of Perinatology and Gynecology, Polish Mother’s Memorial Hospital Research Institute, ul. Rzgowska 281/289, 93-338 Łódź, Poland; Department of Trace Element Analysis by Spectroscopy Method, Faculty of Chemistry, Adam Mickiewicz University in Poznań, ul. Umultowska 89b, 61-614 Poznan, Poland; Department of Education and Obesity Treatment and Metabolic Disorders, University of Medical Sciences, ul. Szamarzewskiego 84, 60-569 Poznan, Poland

**Keywords:** Amniotic fluid, Minerals, Maternal factors, Fetal parameter

## Abstract

The concentrations of various trace elements in amniotic fluid (AF) change over the course of pregnancy, with gestational age and fetus growth. The aim of the present study was to evaluate the concentrations of selected essential and toxic elements in AF and their relations to maternal and fetal parameters. The study was carried out in 39 pregnant women, aged 34.6 ± 4.7 years, between weeks 16 and 26 of gestation. Amniotic fluid samples were obtained during the standard procedure of amniocentesis in high-risk patients for chromosomal abnormalities. An inductively coupled plasma mass spectrometry (ICP-MS) technique was used to determine the levels of Al, As, Ba, Cd, Co, Cr, Cu, Mg, Mn, Ni, Sr, U, and V in AF. Body mass and blood pressure were measured in all the women. The basic parameters of fetal development were also assayed. It was found that the age of the mother, the gender of the fetus, and the week of the pregnancy may affect the concentrations of mineral in the amniotic fluid. Moreover, several significant correlations between the essential and toxic elements and maternal and fetal parameters were observed. In particular, negative and positive correlations between fetal parameters and magnesium and copper levels in AF, respectively, were seen. The present findings demonstrate the association between minerals in AF and fetal development.

## Introduction

During pregnancy, amniotic fluid (AF) surrounds the fetus and is circulated constantly by the baby’s inhalation and exhalation, as well as swallowing and urination, of the fluid.

Maternal plasma—the source of the water in AF—passes through the fetal membranes under hydrostatic and osmotic forces. Through the vessels that develop in the placenta and fetus, water and solute from the maternal plasma pass across the placenta to the fetus and then to the AF. It can thus be assumed that the composition of AF reflects the composition of the fetal plasma [1]. The composition of AF depends on the mother’s nutrition, including essential nutrients, and also on the mother’s exposure to toxic substances. It has been found that proteins or peptides in the amniotic fluid possess potent bioactivity on cellular growth and proliferation and that they modulate the process of embryonic development [2].

The role of trace elements contained in AF is not clear. Because AF is swallowed by the fetus, it has been suggested that this fluid may be an important source of certain trace elements for fetal nutrition. AF is thus considered a valuable marker of prenatal mineral status and exposure to toxic metals.

Only a few investigations to date have evaluated the concentration of minerals in AF. Some studies have determined the essential elements levels in AF, and attempts have been made to establish normal values for the early weeks of pregnancy [1, 3]. In other studies, it was demonstrated that heavy metals may accumulate in AF from a very early stage of gestation; however, little is known about the influence of metals in AF on fetal development or about the long-term effects of this early exposure [4].

However, there is a lack of standards and reference ranges for the concentrations of elements in AF by gestational age. It is not clear what factors determine the concentration of minerals in the AF. Little is known about the relationship between the concentration of elements in AF and fetal development. Accordingly, the aim of this study was to evaluate the concentration of selected essential and toxic elements in AF and their relations with maternal and fetal parameters.

## Material and Methods

The study protocol was approved by the Bioethics Commission at Poznan University of Medical Sciences (approval no. 30/15). Informed consent was obtained from all women. The study was performed in accordance with Helsinki Declaration.

## Subjects

The study was carried out on 39 pregnant women, aged 34.6 ± 4.7 years. The mean gestational age was 20 weeks (range, 16–26 weeks). The inclusion criteria were (1) intrauterine pregnancy, (2) pregnancy without congenital anomalies evaluated in ultrasound, (3) lack of signs and symptoms indicating risk of miscarriage (bleeding, pelvic pain, chorionic hematoma), and (4) informed consent. The exclusion criteria were (1) ectopic or heterotopic pregnancy, (2) multiple pregnancies, (3) positive obstetric history of multiple miscarriages, preterm birth, and fetal growth disorders (growth restriction, macrosomia), (4) observed birth defects or genetic conditions in the current pregnancy or present in the women, (5) use of drugs that affect the mineral balance in the organism, (6) diabetes, (7) infection, (8) gestational hypertension, and (9) maternal exposure to alcohol, cocaine, or tobacco smoke.

The full characteristics for the subjects are presented in Table 1. All subjects were informed of the study’s aims, procedures, and measurement methods, and the individual consent of each patient was obtained.

**Table 1:** Maternal and fetal parameters (*n* = 39)

Parameters	Mean ± SD	Median	Range (min–max)
	Maternal parameters		
Sys (mmHg)	125.1 ± 12.5	126	102–153
Dia (mmHg)	73.4 ± 7.8	74	59–91
Body mass (kg)	68.0 ± 10.3	66	50–90
Age (years)	34.6 ± 4.7	34	24–42
Week of pregnancy	20.3 ± 3.0	20.5	16–26
Fetal parameters			
FHR	148.6 ± 8.9	150	135–178
UAPI	1.41 ± 0.48	1.29	0.63–2.77
HC (mm)	140.0 ± 34.7	132.5	90.9–213
AC (mm)	120.4 ± 29.7	115.1	70.5–179
FL (mm)	24.0 ± 8.2	20.9	12.2–41.0
EFW (g)	232.8 ± 134.5	189.8	84.0–561.1

*Sys* systolic blood pressure, *Dia* diastolic blood pressure, *FHR* fetal heart rate, *UAPI* umbilical artery pulsatility index, *BPD* biparietal diameter, *AC* abdominal circumference, *HC* head circumference, *FL* femur length, *EFW* estimated fetal weight, *SD* standard deviation

## Maternal Parameters Measurements

The body mass was calculated using the measurements of women wearing light clothing with bare feet. Weight was measured to the nearest 0.10 kg accuracy.

Resting arterial blood pressure was measured using regular adult cuffs. The measurements were obtained in a sitting position with the legs uncrossed and the back and arms supported. Blood pressure was measured three times, and an average value was calculated, according to the guidelines of the European Society of Hypertension [5].

## Measurement of Fetal Parameters

Fetal parameters, such as fetal heart rate (FHR), umbilical artery pulsatility index (UAPI), biparietal diameter (BPD), abdominal circumference (AC), head circumference (HC), femur length (FL), and estimated fetal weight (EFW), were measured using ProSound Alpha7 (Aloka Co.) with convex 5–7 MHz probe. The fetal measurements followed the standard protocols for fetal biometry [6].

## Amniotic Fluid

Amniotic fluid (AF) samples were obtained (5 mL) as a residue after genetic analysis as a result for amniocentesis. Samples were collected by transabdominal puncture using plastic syringes free of trace element contamination.

AF samples were centrifuged (3000 rpm/min for 10 min at 4 °C), frozen, and stored at −80 °C.

## Measurement of Minerals in Amniotic Fluid

### Sample Preparation

After collection, amniotic fluid samples were immediately frozen and stored for analysis. Just prior to analysis, the samples were thawed and underwent digestion in a microwave oven model Ethos One (Milestone Srl, Italy). The samples for digestion were prepared as follows: 1 mL of the sample was transferred to the digestion vessels and 0.5 mL of 65 % nitric acid (Merck, Germany) and 0.5 mL of 30 % hydrogen peroxide (Fluka, Germany) were added to each vessel. The microwave oven heating program proceeded in steps: (1) ramp time of 20 min to reach 1500 W, (2) hold time of 30 min at 1500 W, and (3) cooling for 30 min. The temperature during the digestion process was 180 °C. The digested samples were quantitatively transferred into volumetric flasks, which had previously been prepared appropriately, first by soaking in 5 % nitric acid for 24 h and then rinsed with deionized water. The digests were adjusted to 10 mL with deionized water (TKA Smart2Pure, Niederelbert, Germany). In parallel, the procedural blanks, including the same reagents as the samples, were prepared and digested in the same way as the samples in each digestion run.

### Analytical Procedure

An Elan DRC II ICP-MS (PerkinElmerSCIEX, Ontario, Canada) was used to determine Al, As, Ba, Cd, Co, Cr, Cu, Mg, Mn, Ni, Sr, U, and V levels. A cyclonic spray chamber, a concentric glass nebulizer, and a quartz torch with a quartz injector were used. A quadrupole mass analyzer with gold-coated parallel rods was employed, as was an electron multiplier as the detector. Signal intensity was recorded as a counting signal or analog signal, each with its own detection range. The operating conditions for the inductively coupled plasma mass spectrometer (ICP-MS) were as follows: RF power was 1100 W; plasma Ar flow rate was 15 L/min; nebulizer Ar flow rate was 0.86 L/min and auxiliary Ar flow rate was 1.2 L/min; and lens voltage was 7.0–9.5 V. Argon with a purity of 99.999 % was used as a nebulizer, auxiliary, and plasma gas (Linde Gaz, Poland). A mixed standard solution (Multielement Calibration Standard 3, Atomic Spectroscopy Standard, PerkinElmer Pure) containing analyzed elements at a concentration of 10 mg L^−1^ was used to construct calibration curves. The calibration curves for the elements were constructed in the range of 0.01 to 50 μg L^−1^. The isotopes of ^45^Sc, ^74^Ge, ^103^Rh, and ^159^Tb prepared from individual solutions with a concentration of 1000 mg L^−1^ were applied as internal standards in order to eliminate nonspectral interferences (ICP Standard CertiPUR, Merck, Germany). In turn, spectral interference was eliminated through the use of a dynamic reaction cell (DRC). As the DRC reaction gas, high-purity ammonia (99.999 %) was used. Deionized water was used throughout the experiment [7].

### Figures of Merit and Analytical Performance

After calibration, and also during the analysis, measurements were controlled by analysis of standard solutions at concentrations of 1 or 5 μg L^−1^ and certified reference materials after each batch of ten samples. Calibration curves for all elements proved to be linear over the whole concentration range, which resulted in good correlation coefficients of at least 0.999. In order to ensure the reliability of the applied analytical procedure, analysis of the certified reference material is highly recommended. The accuracy of the method for the elements under investigation was evaluated by analyzing ten samples of the certified reference material of river water SLRS-5 and fortified lake water TM 27.3. The obtained values are presented in Table 2. Within day precision was calculated as the relative standard deviation and expressed in a percentage as the variation coefficient. The precision values for all the tested elements fell in the range from 1.2 to 22.2 % for SLRS-5; however, for TM 27.3, the values were found to be in the 0.5–4.0 % range. The limits of detection (LOD) were calculated as three standard deviations from the procedural blank samples. LOQ values were calculated as three times of the LOD values. LOD and LOQ values are gathered in Table 3.

**Table 2:** Results of the measurements (*n* = 10) of certified reference materials: SLRS-5 and TM 27.3 given with certified values

Analyte	River water SLRS-5			Fortified lake water TM 27.3		
	Measured value (μg L^−1^)	Certified value (μg L^−1^)	Accuracy (%)	Measured value (μg L^−1^)	Certified value (μg L^−1^)	Accuracy (%)
Al	46.84 ± 0.65	49.5 ± 5.0	95	42.8 ± 1.7	44.4 ± 5.1	96
As	0.448 ± 0.039	0.413 ± 0.039	109	2.258 ± 0.030	2.15 ± 0.30	105
Ba	14.6 ± 1.3	14.0 ± 0.5	104	15.96 ± 0.21	14.9 ± 1.1	107
Cd	0.0095 ± 0.0021	0.0060 ± 0.0014	159	1.110 ± 0.014	1.05 ± 0.12	106
Co	0.0607 ± 0.0028	0.05^a^	121	1.888 ± 0.020	2.05 ± 0.18	92
Cr	0.289 ± 0.044	0.208 ± 0.023	139	1.953 ± 0.010	1.73 ± 0.33	113
Cu	17.07 ± 0.54	17.4 ± 1.3	98	6.01 ± 0.12	6.16 ± 0.61	98
Mg	2752 ± 46	2540 ± 160	108	–	–	–
Mn	4.12 ± 0.32	4.33 ± 0.18	95	2.232 ± 0.016	2.27 ± 0.35	98
Ni	0.589 ± 0.016	0.476 ± 0.064	124	2.396 ± 0.070	2.44 ± 0.53	98
Sr	52.40 ± 0.65	53.6 ± 1.3	98	98.4 ± 1.4	105 ± 6	94
U	0.0908 ± 0.0090	0.093 ± 0.006	98	2.094 ± 0.075	2.03 ± 0.19	103
V	0.360 ± 0.049	0.317 ± 0.033	114	2.170 ± 0.042	2.18 ± 0.25	100

^a^information value

**Table 3:** LOD and LOQ values

	LOD (μg L^−1^)	LOQ (μg L^−1^)
Al	6	18
As	0.04	0.12
Ba	0.25	0.75
Cd	0.004	0.012
Co	0.004	0.012
Cr	0.2	0.6
Cu	0.4	1.2
Mg	5	15
Mn	0.3	0.9
Ni	0.1	0.3
Sr	0.3	0.9
U	0.009	0.027
V	0.04	0.12

## Statistical Analysis

A detailed statistical analysis was performed with Statistica 10 for Windows. The normality of the variables’ distribution was verified using the Shapiro–Wilk test. The Mann–Whitney *U* test was used to compare differences between groups for all the studied parameters. Simple associations between parameters were calculated as the Spearman coefficient of correlation. The level of statistical significance was set to *p* < 0.05.

## Results

The mean, range, and median concentrations of the essential and toxic minerals in AF are shown in Table 4.

**Table 4:** Concentrations of mineral in amniotic fluid (*n* = 39)

Minerals	Mg	Al	Cu	Co	Ni	As	Sr	Cd	Ba	V	U	Cr	Mn
	μgL^−1^												
Mean ± SD	8009.7 ± 1952.1	158.6 ± 135.5	106.7 ± 53.6	0.23 ± 0.10	4.31 ± 4.02	4.24 ± 2.10	26.4 ± 16.8	0.07 ± 0.06	7.74 ± 12.3	8.20 ± 3.27	0.039 ± 0.03	1.96 ± 2.26	5.78 ± 5.01
Median	8287	114.8	91.5	0.21	3.67	3.85	23.7	0.06	0.25	8.17	0.027	0.83	4.75
Range (min–max)	3618–11,634	9.9–660.0	37.6–359.2	0.09–0.57	0.18–22.28	0.95–10.3	11.5–109.8	0.007–0.282	0.15–60.7	1.93–15.9	0.012–0.122	0.20–8.12	0.83–28.8

*SD* standard deviation, *min* minimum value, *max* maximum value

In this study, it was presumed that the factors most likely to influence the mineral concentrations in amniotic fluid are as follows: age of mothers, week of the pregnancy, and gender of the fetus.

It has been postulated that pregnancy in women over 35 years of age is associated with an increased risk of malformations. For this reason, subjects in this study were divided into two groups according to age: those less than 35 years old (*n* = 17) and those 35 years or older (*n* = 22). It was found that the concentration of magnesium was significantly lower and that the levels of nickel, cadmium, barium, and chromium were markedly higher in the AF of older women (Table 5).

**Table 5:** Concentrations of mineral in amniotic fluid according to the maternal age (μgL^−1^)

Minerals	Mg	Al	Cu	Co	Ni	As	Sr	Cd	Ba	V	U	Cr	Mn
< 35-year-old women (*n* = 17)													
Mean ± SD	8783.9 ± 1395.2	188.7 ± 185.4	102.4 ± 33.0	0.25 ± 0.08	4.21 ± 5.88	4.40 ± 2.23	32.0 ± 24.8	0.06 ± 0.07	2.16 ± 5.05	7.96 ± 3.70	0.05 ± 0.03	0.61 ± 1.09	6.95 ± 7.67
Median	8723.0^a^	114.7	92.8	0.25	2.32^b^	4.04	23.7	0.04^b^	0.25^b^	8.58	0.04	0.20^b^	4.24
≥35-year-old women (*n* = 22)													
Mean ± SD	7471.2 ± 2125.0	145.0 ± 108.3	109.7 ± 64.8	0.22 ± 0.12	4.37 ± 2.30	4.14 ± 2.05	22.4 ± 5.5	0.08 ± 0.05	11.8 ± 14.4	8.38 ± 3.01	0.04 ± 0.03	2.91 ± 2.39	5.11 ± 2.57
Median	7501^b^	114.8	90.3	0.20	3.98^a^	3.73	23.7	0.08^a^	10.3^a^	7.95	0.02	2.31^a^	4.86

*SD* standard deviation

^a, b^significantly different, Mann–Whitney’s test, *p* < 0.05

The samples were also divided into two different groups according to the gender of the fetus. A significant difference was found in the vanadium concentration in AF, with higher levels of vanadium shown in male fetuses than in female fetuses (Table 6).

**Table 6:** Concentrations of mineral in amniotic fluid according to the gender of the fetus (μgL^−1^)

Minerals	Mg	Al	Cu	Co	Ni	As	Sr	Cd	Ba	V	U	Cr	Mn
Male (*n* = 20)													
Mean ± SD	7663.5 ± 2156.1	179.3 ± 147.2	104.1 ± 37.7	0.23 ± 0.11	4.27 ± 4.84	4.35 ± 2.19	25.3 ± 20.8	0.06 ± 0.06	6.88 ± 9.48	8.62 ± 3.45	0.03 ± 0.02	2.18 ± 2.46	6.61 ± 6.12
Median	8332.5	115.4	91.9	0.21	3.85	3.94	22.5	0.06	0.25	8.8^a^	0.03	1.22	4.64
Female (*n* = 19)													
Mean ± SD	8288.5 ± 1675.5	107.1 ± 79.4	107.7 ± 79.1	0.22 ± 0.07	3.61 ± 2.79	3.74 ± 1.94	26.01 ± 6.89	0.07 ± 0.04	5.08 ± 8.26	6.58 ± 2.26	0.05 ± 0.04	1.25 ± 1.48	4.26 ± 2.78
Median	8118	105.8	85.4	0.20	2.94	3.44	25.1	0.06	0.25	6.26^b^	0.03	0.63	4.23

*SD* standard deviation

^a, b^significantly different, Mann–Whitney’s test, *p* < 0.05

The amniotic fluid samples were divided into two additional groups according to the week of pregnancy. The one of the group was between 16 and 20 week of gestation and the second group was between 21 and 26 week of gestation. The markedly higher concentration of copper and lower level of magnesium in the samples with longer period of pregnancy was observed (Table 7).

**Table 7:** Concentration of minerals in amniotic fluid according to week of the pregnancy (μgL^−1^)

Minerals	Mg	Al	Cu	Co	Ni	As	Sr	Cd	Ba	V	U	Cr	Mn
16–20 (*n* = 19)													
Mean ± SD	8648.33 ± 1913.16	110.94 ± 90.13	75.05 ± 17.03	0.21 ± 0.06	3.10 ± 3.03	4.14 ± 2.00	20.61 ± 6.73	0.05 ± 0.04	3.70 ± 7.87	7.90 ± 2.89	0.04 ± 0.03	1.16 ± 1.73	3.85 ± 2.35
Median	9383^a^	96.4	82.64^a^	0.22	2.54	3.91	21.31	0.04	0.25	8.70	0.03	0.2	3.57
21–26 (*n* = 20)													
Mean ± SD	7581.09 ± 1887.31	160.46 ± 141.79	115.16 ± 60.13	0.23 ± 0.11	4.77 ± 4.76	4.02 ± 2.07	29.39 ± 20.90	0.07 ± 0.06	6.40 ± 8.92	7.52 ± 3.11	0.04 ± 0.03	1.87 ± 2.19	6.52 ± 6.03
Median	8287^b^	114.79	97.55^b^	0.20	3.96	3.6	23.65	0.06	0.25	7.02	0.03	1.44	5.65

^a, b^significantly different, Mann–Whitney’s test, *p* < 0.01

Table 8 shows the correlation between mineral concentrations in AF and selected maternal and fetal parameters. The maternal and fetal parameters are summarized in Table 1. Significant positive correlations between systolic blood pressure and the level of uranium and manganese in AF, and between diastolic blood pressure and the levels of nickel, strontium, cadmium, and manganese, were found. Moreover, a markedly negative correlation between the current body mass of women and the level of magnesium in AF was observed. Furthermore, there was a significant correlation between fetal parameters and magnesium and copper levels in AF. Significant positive correlations were seen between magnesium and umbilical artery pulsatility index (UAPI), copper and biparietal diameter (BPD), copper and abdominal circumference (AC), copper and head circumference (HC), copper and femur length (FL), and copper and estimated fetal weight (EFW) (Table 7). Moreover, markedly negative correlations between magnesium and BPD, HC, FL, and EFW were observed.

**Table 8:** Correlation between mineral levels in amniotic fluid and maternal and fetal parameters

Parameters	Mg	Al	Cu	Co	Ni	As	Sr	Cd	Ba	V	U	Cr	Mn
Maternal parameters													
Sys	−0.13	0.35	0.11	0.11	0.17	0.15	0.19	0.26	0.12	0.12	0.39*	−0.01	0.45*
Dia	−0.05	0.02	−0.06	0.10	0.36*	−0.10	0.35*	0.46*	0.00	−0.11	0.15	0.14	0.37*
Body mass	−0.45*	−0.04	−0.02	−0.25	0.07	−0.29	−0.31	0.06	0.29	−0.31	−0.21	0.15	0.04
Fetal parameters													
FHR	0.05	−0.15	0.09	−0.32	0.09	0.05	−0.15	−0.05	−0.12	0.27	−0.03	0.07	0.18
UAPI	0.40*	0.07	−0.27	−0.03	−0.09	0.02	0.14	0.00	−0.20	−0.10	0.12	−0.15	−0.09
BPD	−0.39*	0.15	0.41*	0.15	0.16	−0.08	0.12	0.27	0.29	−0.08	0.09	0.25	0.20
HC	−0.38*	0.13	0.41*	0.14	0.15	−0.11	0.12	0.25	0.30	−0.12	0.11	0.24	0.16
AC	−0.27	0.09	0.40*	0.15	0.11	−0.08	0.11	0.21	0.19	−0.08	0.02	0.16	0.14
FL	−0.40*	0.14	0.41*	0.14	0.22	−0.12	0.15	0.29	0.28	−0.13	0.03	0.27	0.25
EFW	−0.34*	0.12	0.41*	0.13	0.17	−0.09	0.11	0.25	0.22	−0.09	0.00	0.24	0.20

*Sys* systolic blood pressure, *Dia* diastolic blood pressure, *FHR* fetal heart rate, *UAPI* umbilical artery pulsatility index, *BPD* biparietal diameter, *AC* abdominal circumference, *HC* head circumference, *FL* femur length, *EFW* estimated fetal weight

*Spearman correlation (*R*), *p* < 0.05

## Discussion

The concentration of minerals in amniotic fluid was affected by maternal age, week of gestation, and gender of fetus. This is a novel finding of our study. We also showed that the level of minerals in AF correlates with some maternal and fetal parameters. To the best of our knowledge, this is the first study on pregnant women to demonstrate these relationships.

The range of concentration of some minerals in AF in this study—especially copper, strontium, cobalt, manganese, and barium—is in agreement with the results of other reports [1, 4]. However, some other authors have observed lower levels of aluminum, copper, nickel, strontium, and vanadium and higher levels of barium and magnesium in amniotic fluid than in this study [2, 4, 8]. There are several factors that may affect the differences in the results obtained in various studies, such as age of the mother, week of pregnancy, and the analytical methods used. In this study, it was found that the concentration of metals such as nickel, cadmium, and barium increased in AF with maternal age. It is possible that, in older mothers, the period of exposure to metals was longer, and hence, the accumulation of these minerals in the body of the mothers and their passage into the AF was greater [9]. Higher concentrations of metal in AF may suggest increased risks of abnormal fetal development, because the fetus inhales and swallows amniotic fluid at a turnover rate of one third of its volume every hour [8]. In this study, it was observed that lower levels of magnesium were found in the amniotic fluid of older mothers. Low magnesium levels in AF are associated with complications of pregnancy such as preeclampsia and diabetes [3], and it is known that women with advanced maternal age are at high risk of developing these diseases [10–12]. Moreover, the concentration of some minerals (including magnesium) in women’s bodies may depend on age. It was found that the concentration of magnesium in the hair of women of reproductive age was significantly lower in subjects in the 31–40 age range than in younger or older subjects [13].

In the later weeks of gestation, the concentration of copper in AF was significantly higher. This may be caused by longer time of maternal exposure to this element from food, water, and environment, and the increased secretion of copper in fetal urine. It is known that the absorption of copper rises during pregnancy due to the increasing need for maternal copper-containing enzymes, and copper is also critical in fetal development [1]. In this study, we observed the positive correlations between copper concentration in AF and fetal biometric parameters. Similar concentrations of copper in the AF of women of similar age and in similar weeks of pregnancy have been reported by other authors [14]. The increase in the level of copper and other minerals in AF as pregnancy progressed was also observed in experimental models in pigs [15]. Because AF is swallowed by the fetus, Richards [15] suggested that this fluid may be an important source of copper for fetal nutrition. Increases in copper concentration in AF may reflect an increasing level of this mineral in the mother’s blood during pregnancy [16]. It was found that the concentration of trace elements in AF linearly correlates with maternal serum [16]. Other authors have found that zinc in human AF increases during pregnancy and that the most significant period for this rise is the third trimester [17].

In this study, the concentration of magnesium was lower in the samples of amniotic fluid with longer gestational age in the second trimester. These results were confirmed by the study of Facchinetti et al. [18]. It is known that magnesium plays an important role in pregnancy and fetal development. The fall in levels of magnesium over the course of pregnancy can attest to the fact that this essential mineral is used for building the body and metabolism in the fetus. This finding is very important, because magnesium deficiency may be a major factor of pathogenesis in complicated pregnancies and also may cause metabolic problems extending into adult life [19, 20].

In several studies, fetal gender was seen to relate to the concentration of some AF components, such as cytokines, hormones, and minerals [21–23]. Tzschoppe et al. [23] found significantly higher concentrations of potassium, magnesium, and phosphate in the AF of male fetuses. In this study, higher levels of vanadium were seen in the amniotic fluid of male fetuses than of female fetuses. These results may point to sex-specific differences in physiological or hormonal changes in fetuses. Confirmation of such differences is provided by one study that showed that male fetal gender is associated with higher levels of proinflammatory cytokines and angiogenic factors in the mother’ s blood than female fetal gender [24]. It is known that vanadium is a factor associated with the process of angiogenesis [25], and this may also partly explain the present study’s results.

It was found that high blood pressure in mothers correlated with higher concentration of nickel, strontium, cadmium, uranium, and manganese in AF. Relationships between the levels of these metals and high blood pressure in different populations (including in pregnant women) have been demonstrated in several studies [26–30]. Since amniotic fluid is swallowed by the fetus, higher levels of metals in this fluid may lead to an increased risk of hypertension in the child.

The significant correlations that were observed between minerals in the AF and the fetal parameters are partially confirmed by the results for gestational age (Tables 7 and 8). As pregnancy proceeded, the concentration of copper in AF increased and magnesium concentration decreased. Moreover, fetal growth parameters such as BPD, AC, HC, FL, and EFW are negatively correlated with the concentration of magnesium and positively correlated with the level of copper in the AF.

In this study, a significant positive correlation was found between the magnesium level in AF and umbilical artery pulsatility index (UAPI). The association between these parameters was also demonstrated in other studies [31–33]. Moreover, it was observed that the UAPI value, like the magnesium concentration, decreases with gestational age [18, 31].

This study has some limitations. In particular, the size of the group was small, and so this research is rather a pilot study. Due to the small group size, we could not attempt to establish reference values for the concentrations of minerals in the amniotic fluid at given gestational ages. We analyzed only certain minerals in the amniotic fluid, but not in the mother’s blood or in the umbilical cord blood, which would allow broader conclusions about the association between mineral status and fetal development. Moreover, the daily intake and eating habits of the mothers’ were not included in this study, and therefore, it was not possible to determine the influence of mothers’ diet on minerals concentration in the amniotic fluid samples.

In conclusion, our results suggest a relationship between mineral level in AF and fetal development. These findings can present an additional opportunity for diagnosing fetal development. Further investigations with a larger group of pregnant women is needed to explain the mechanism of this association and to determine of the reference values for mineral levels in the amniotic fluid at various stages of pregnancy.
